# Bibliometric and visual analysis in the field of electroacupuncture’s analgesia and regulation on negative emotion from 2014 to 2024

**DOI:** 10.3389/fneur.2025.1502657

**Published:** 2025-02-10

**Authors:** Xubo Huang, Jiajie Gao, Yuxin Ding, Jiali Wang, Junfan Fang, Jianqiao Fang, Junying Du

**Affiliations:** ^1^The Third School of Clinical Medicine, Zhejiang Chinese Medical University, Hangzhou, China; ^2^Department of Neurobiology and Acupuncture Research, The Third School of Clinical Medicine, Zhejiang Chinese Medical University, Hangzhou, China

**Keywords:** electroacupuncture, analgesia, emotional regulation, chronic pain, bibliometric analysis

## Abstract

**Introduction:**

This bibliometric study systematically analyzes the research landscape of electroacupuncture (EA), focusing on its applications in pain relief and emotional regulation from 2014 to 2024. EA, a contemporary adaptation of traditional acupuncture, has gained significant attention for its potential therapeutic benefits in managing chronic pain and mood disorders.

**Methods:**

Using the Web of Science Core Collection as the primary data source, we identified 537 articles related to EA’s therapeutic effects. The analysis was conducted using bibliometric tools such as VOSviewer and CiteSpace to visualize publication trends, research hotspots, and collaborative networks.

**Results:**

The study highlights a significant upward trend in research output, with a marked increase in publications from 2019 onwards. China emerged as the leading contributor, accounting for over 60% of the total research output, followed by the United States and South Korea. Key institutions, such as Zhejiang Chinese Medical University and Shanghai University of Traditional Chinese Medicine, have made substantial contributions, emphasizing the importance of traditional Chinese medicine in this research area. Major research themes include the modulation of neurotransmitter systems, the role of endogenous opioids, and the impact of EA on chronic pain and mood disorders. Collaborative networks between countries and institutions are mapped, revealing the centrality of Chinese and American research partnerships.

**Discussion:**

This comprehensive analysis outlines the current state of EA research and identifies gaps and opportunities for future studies, particularly in understanding the mechanistic pathways of EA and its integration into mainstream medical practices. The findings provide a roadmap for enhancing the therapeutic applications of EA and underscore its potential in managing complex conditions involving both physical and emotional components.

## 1 Introduction

Electroacupuncture (EA), a contemporary adaptation of traditional acupuncture, has garnered increasing attention in recent years due to its potential therapeutic benefits in both pain management and emotional regulation (1). As a method that integrates the principles of classical acupuncture with the application of electrical currents to acupuncture points, EA has been investigated extensively in both clinical and experimental settings. This hybrid technique is believed to enhance the effects of traditional acupuncture by delivering consistent and controllable electrical stimuli, thereby potentially improving treatment outcomes for a variety of conditions, including chronic pain, anxiety, and depression (2, 3).

Pain, particularly chronic pain, is a complex and multifaceted phenomenon that involves not only physical sensations but also significant emotional and cognitive components (4). The interplay between chronic pain and emotional states such as anxiety and depression has been well-documented, with research indicating that these emotional factors can intensify the perception of pain, leading to a debilitating cycle that adversely affects patients’ quality of life (5). Traditional acupuncture has been employed for millennia in Eastern medicine to address both physical and psychological ailments, with its mechanisms of action often attributed to the regulation of the body’s energy flow, or “Qi,” through specific meridians (6). However, the advent of EA has introduced a new dimension to this practice, offering a more precise and measurable intervention that may hold greater efficacy in certain cases.

The mechanisms underlying EA’s analgesic and mood-regulating effects are an area of active research (7, 8). Studies have suggested that EA can modulate the central and peripheral nervous systems, influencing neurotransmitter release, altering pain signal processing, and promoting the release of endogenous opioids (9). These effects are thought to contribute to its ability to alleviate pain and improve mood, making it a promising therapeutic option for conditions where these two aspects are closely linked, such as in fibromyalgia, osteoarthritis, and various neuropathic pain disorders (10–12).

In parallel with the clinical and mechanistic research, the field of bibliometrics has emerged as a powerful tool for understanding the development and dissemination of scientific knowledge. Bibliometric analysis allows researchers to quantitatively assess the evolution of research fields, identifying key contributors, influential publications, and emerging trends over time. Despite the growing volume of research on EA, particularly in relation to its dual role in pain relief and emotional regulation, there remains a paucity of comprehensive bibliometric studies that analyze this specific intersection. Notably, this study represents the first systematic review to focus on both the analgesic effects and emotional regulation in EA research, offering a novel lens through which to explore the broader implications of EA in clinical practice (13–17). A bibliometric study in this area would not only highlight the current state of research but also provide strategic insights for future studies, potentially guiding more targeted and impactful research efforts. This study, therefore, aims to conduct a thorough bibliometric analysis of the literature surrounding EA’s role in analgesia and emotional regulation. The results are expected to offer a detailed overview of how this field has evolved over time, the current research hotspots, and potential future directions, thereby contributing valuable knowledge to both the scientific community and clinical practice.

## 2 Materials and methods

### 2.1 Search strategy

**Figure 1:**
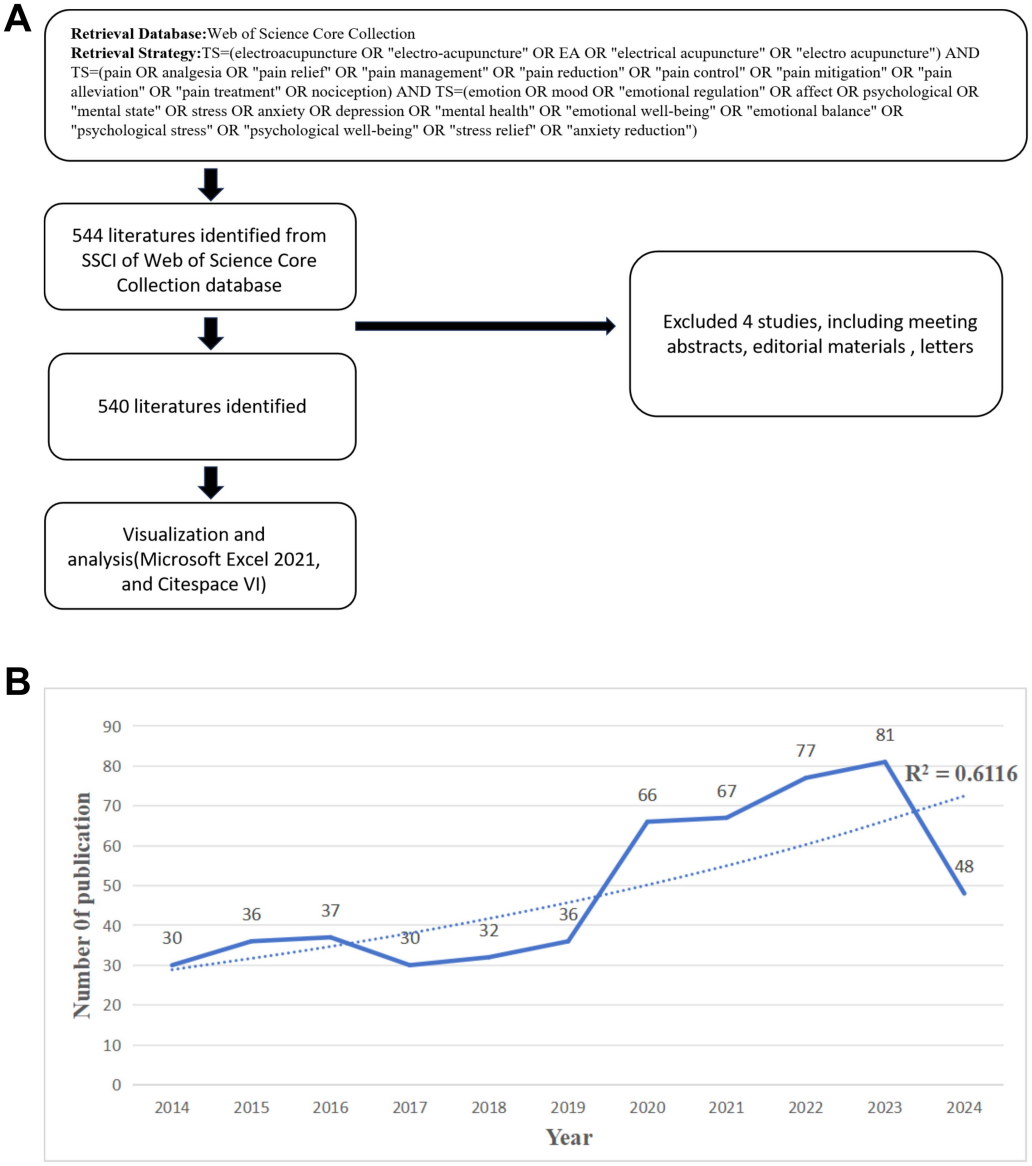
Search strategies and trends in the number of articles published. **(A)** Search strategy and selection process. **(B)** Trends in the growth of publications.

The data for this bibliometric analysis were extracted from the Web of Science Core Collection (WoSCC) database (Figure 1A). The WoSCC was selected due to its comprehensive coverage of high-quality, peer-reviewed articles across multiple disciplines, as well as its rigorous indexing standards. Although other databases such as Scopus and PubMed could also provide valuable data, WoSCC was chosen for this study to ensure consistency and reliability in the data retrieval process, given its established reputation in bibliometric research. The search was performed using the following query: TS (Topic) = (electroacupuncture OR “electro-acupuncture” OR EA OR “electrical acupuncture” OR “electro acupuncture”) AND TS = (pain OR analgesia OR “pain relief” OR “pain management” OR “pain reduction” OR “pain control” OR “pain mitigation” OR “pain alleviation” OR “pain treatment” OR nociception) AND TS = (emotion OR mood OR “emotional regulation” OR affect OR psychological OR “mental state” OR stress OR anxiety OR depression OR “mental health” OR “emotional well-being” OR “emotional balance” OR “psychological stress” OR “psychological well-being” OR “stress relief” OR “anxiety reduction”).

### 2.2 Data collection and analysis

The search was limited to articles and reviews published in English between January 1, 2014, and August 1, 2024. Conference abstracts, editorials, letters, and book chapters were excluded to focus on original, peer-reviewed research contributions.

This search yielded a total of 540 articles. All searches and data downloads were conducted on August 1, 2024, ensuring that the results reflected the most current state of research at the time. The retrieved data were then processed and imported into bibliometric software tools include VOSviewer and CiteSpace, enabling the visualization and analysis of publication trends, research hotspots, and collaborative networks within the context of EA’s effects on pain and emotional well-being.

## 3 Results

### 3.1 Annual publication volume and trends

The analysis of annual publication volume on EA for pain relief and emotion alleviation from 2014 to 2024 reveals a significant upward trend, particularly in the last 5 years (Figure 1B).

Between 2014 and 2018, the number of publications remained relatively stable, with minor fluctuations. The annual output ranged from 30 to 37 publications, indicating a consistent but moderate level of research activity. This period likely represents a phase where foundational research was being established, setting the stage for subsequent growth. A notable increase in publications occurred from 2019 onwards. In 2019, the publication volume reached 36, followed by a substantial rise to 66 publications in 2020. This growth trend continued, with 67 publications in 2021 and a further increase to 77 in 2022. The peak in publication volume was observed in 2023, with a total of 81 publications, marking the highest annual output within the analyzed period. In 2024, there appears to be a decline in publication numbers, with only 48 publications recorded. However, this apparent decrease is due to the fact that the data collection for this bibliometric analysis only included articles published up to August 1, 2024. Given the strong upward trend observed in previous years, it is likely that the total number of publications for 2024 will surpass that of 2023 once the entire year’s data is available.

Overall, the data demonstrate a clear trend of increasing research activity, underscoring the growing importance and recognition of EA as a viable therapeutic modality. The surge in recent years highlights the expanding scope of investigation and the field’s integration into broader medical and scientific discourse.

### 3.2 Research countries/regions and their relationships

**Figure 2:**
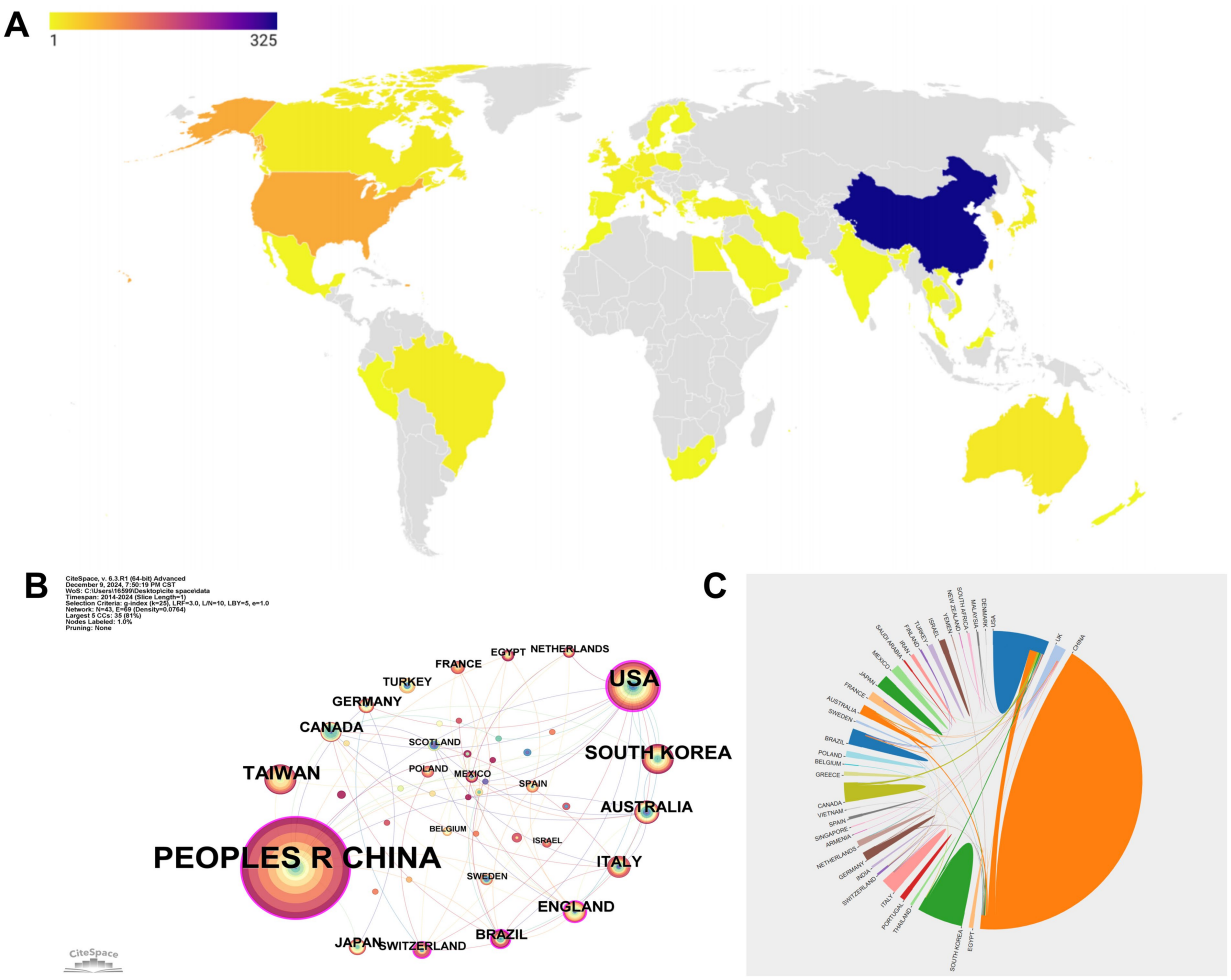
National publications and international cooperation. **(A)** Number of articles published by country. **(B)** Cooperation map of countries/regions. The size of the nodes represents the total output of each country, with larger nodes indicating greater research contributions. The thickness of the lines connecting the nodes reflects the strength of the collaborative relationships, with thicker lines signifying stronger, more frequent partnerships. **(C)** International scientific collaboration chord diagram. The size of each node directly corresponds to the volume of publications from that country, while the lines connecting the nodes represent the frequency of collaborations between countries.

In total (Figure 2A), 52 countries/regions have contributed to the research, reflecting a wide global distribution of efforts. China’s dominance in the research landscape is unmistakable, with a remarkable contribution of 325 publications, far outpacing the United States, which produced 77, and South Korea and Taiwan, with 33 and 32 publications, respectively. This substantial output underscores China’s central role in driving research forward, positioning it as the leading force in this field. While the United States and other countries like Australia, with 16 publications, and Italy, with 15, play significant roles, they still trail behind China’s overwhelming influence. These figures not only highlight China’s leadership but also illustrate the varying levels of contributions from different nations across the globe.

The interconnectedness of these research efforts is further illustrated by the global collaboration network (Figure 2B). In this diagram, the People’s Republic of China and the United States are prominently featured, not only leading in research output but also in forming robust partnerships with other countries. This network highlights not only the volume of research produced by these countries, but also their pivotal role in fostering international cooperation. Through these collaborations, a more interconnected and synergistic global research community has emerged, driving advancements in the field.

In the country collaboration chord diagram (Figure 2C), China and the United States dominate the diagram with the largest nodes, reflecting their substantial research output. The extensive lines linking these two countries to others, such as England, Canada, and Brazil, illustrate the high frequency of their international collaborations. Even nations with smaller publication volumes, such as Turkey, Switzerland, and Egypt, play significant roles in the global research community, as indicated by their connections in the diagram. These collaborations are crucial, as they not only enhance the global impact of individual research efforts but also foster a more integrated and cooperative international research environment, where knowledge is shared and advanced collectively.

### 3.3 Institutions

**Figure 3:**
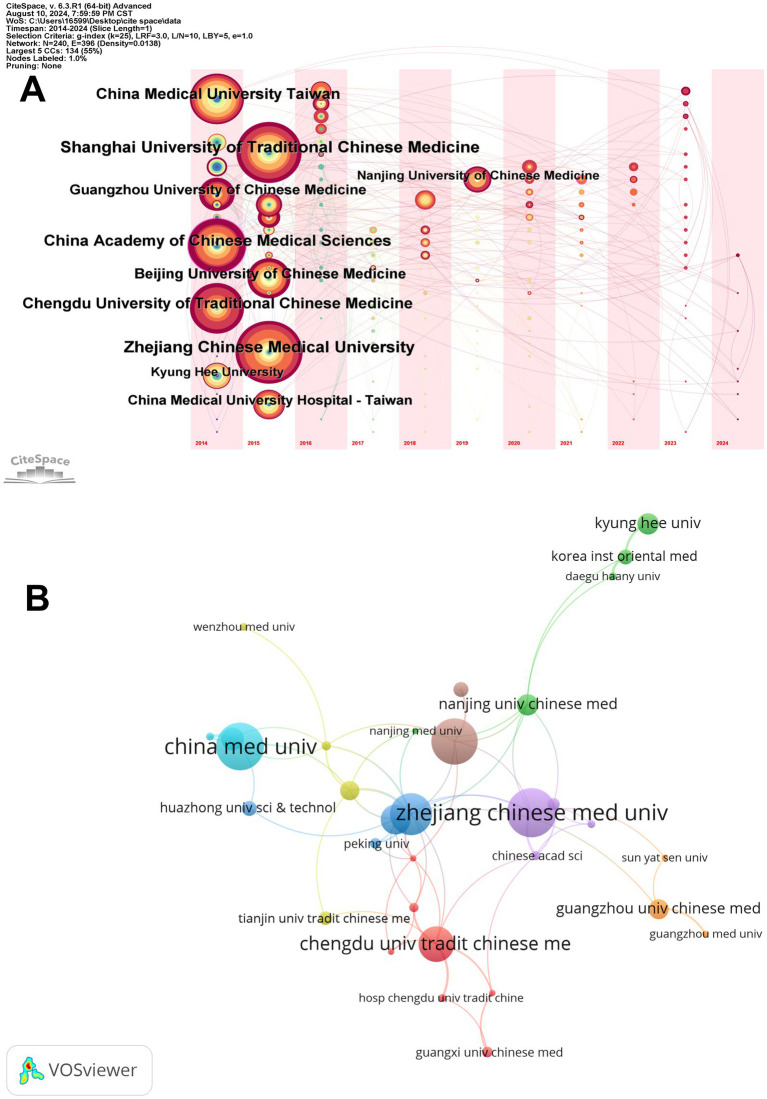
Institutional publications and collaborations. **(A)** Temporal overlay of the institution’s cooperative network. Each node represents an institution, and the size of the node correlates directly with the number of papers produced by that institution. **(B)** The institutional collaboration map. The size of each node corresponds to the number of papers produced by an institution, while the thickness of the connecting lines represents the strength of the collaborative relationships between these institutions.

The CiteSpace timeline overlay map (Figure 3A) visually illustrates the research contributions of various institutions in the specific areas of EA analgesia and emotional relief within traditional Chinese medicine over time. Larger nodes, such as those for Zhejiang Chinese Medical University (36 papers) and Shanghai University of Traditional Chinese Medicine (35 papers), indicate these institutions’ significant contributions to the field. The timeline shows how these institutions have maintained or increased their research output over the years, with consistent activity highlighted by the presence and size of nodes in consecutive years. The evolution of these nodes over time reflects the sustained and growing influence of these key institutions in traditional Chinese medicine research. Additionally, the map allows us to trace the emergence and growth of other institutions, such as Kyung Hee University (15 papers) and China Medical University in Taiwan (29 papers), whose nodes have expanded in recent years, indicating their increasing prominence in the field.

The second image, created using VOSviewer, illustrates a collaboration network among institutions that have published more than five papers (Figure 3B). Notably, institutions such as Zhejiang Chinese Medical University and Shanghai University of Traditional Chinese Medicine are central in this network, highlighting their significant contributions and extensive collaborations. The dense web of connections, particularly among Chinese institutions, underscores the importance of cooperation in driving research forward.

### 3.4 Authors and cited authors

**Figure 4:**
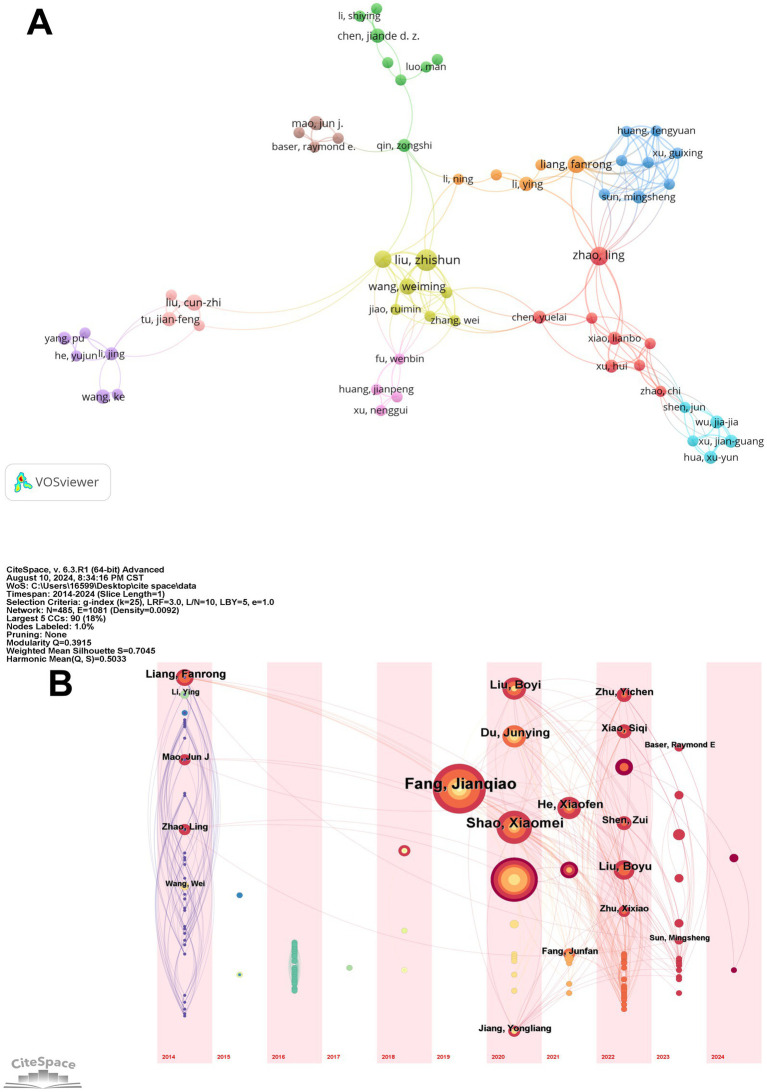
Author publications and collaborations. **(A)** The author collaboration network. **(B)** Temporal overlay of the author’s cooperative network.

We identified and analyzed 485 authors, and the author collaboration network is visualized in Figure 4A. This network highlights the collaborative relationships among these authors, with Fang Jianqiao emerging as the most influential figure. His central position in the network, coupled with the size of his node, indicates his extensive collaborations and significant impact within the field. Notably, Fang Jianqiao’s close collaborations with researchers such as Du Junying and Shen Zui further solidify his role as a key connector in the scholarly community. In contrast, smaller clusters, including those led by Sun Haiju and Liu Boyi, represent more specialized research groups that focus on particular aspects of acupuncture, emphasizing the multidisciplinary nature of the field.

The temporal overlay of authors’ contributions, depicted in Figure 4B, provides further insights into the evolving influence of key researchers over time. This visualization reveals that while Fang Jianqiao has maintained a consistent and prominent presence in the field, other authors like He Xiaofen and Shao Xiaomei have seen a significant rise in their influence, particularly in recent years. The color progression from purple to yellow indicates the timeline of their publications, showing that their research has become increasingly central to the field since 2020. This pattern suggests that these authors have introduced innovative ideas or methodologies that have resonated with the research community, marking them as emerging leaders.

The co-citation analysis underscores the significant influence of key authors in the field of acupuncture for pain relief and emotional regulation, highlighting their specific areas of expertise. Han Jisheng, who tops the list with 93 citations and a centrality of 0.15, is renowned for his pioneering work on the neurobiological mechanisms of EA, particularly its role in modulating pain through endogenous opioids (18). Zhang Ruixin, with 73 citations and a centrality of 0.10, has made substantial contributions to understanding EA’s effects on cancer pain, utilizing rat models to explore its mechanisms (19). Andrew J Vickers, recognized for his contributions to clinical trials and meta-analyses, has a centrality of 0.16, with his research critically evaluating the efficacy of EA in various clinical settings (20).

### 3.5 Journals and co-cited journals

The analysis of publications (Table 1) contributing to the field of EA for pain relief and emotional regulation reveals a concentration of publications in mid-impact journals, with a notable presence in the Q3 quartile. Evidence-Based Complementary and Alternative Medicine, which ranks first in publication volume with 32 papers (5.93%), and Trials, with 27 papers (5.00%), both fall within the Q3 quartile. This dominance of Q3 journals suggests that while the topic of EA is gaining traction, it may still be underrepresented in higher-impact journals. This could be due to the specialized nature of EA research, which may not yet appeal to broader, more competitive medical journals. Additionally, only a small percentage of the top 10 journals, such as BMC Complementary and Alternative Medicine and American Journal of Chinese Medicine, are classified in the Q1 quartile, indicating a limited reach within the highest-impact segment of scientific publications. This distribution highlights a potential challenge for the field in gaining wider acceptance and recognition within the mainstream medical community.

**Table 1:** Top 10 most published journals.

Rank	Journal	Count	%of 540	JIF (2024)	Quartile in category (2024)
1	*EVIDENCE-BASED COMPLEMENTARY AND ALTERNATIVE MEDICINE*	32	5.93%	2.650	Q3
2	*TRIALS*	27	5.00%	1.996	Q3
3	*JOURNAL OF PAIN RESEARCH*	22	4.07%	2.533	Q2
4	*MEDICINE*	22	4.07%	1.345	Q3
5	*ACUPUNCTURE IN MEDICINE*	16	2.96%	2.357	Q3
6	*FRONTIERS IN NEUROLOGY*	15	2.78%	2.741	Q2
7	*BMC COMPLEMENTARY AND ALTERNATIVE MEDICINE*	12	2.22%	4.782	Q1
8	*PLOS ONE*	11	2.04%	2.928	Q2
9	*FRONTIERS IN NEUROSCIENCE*	10	1.85%	3.202	Q2
10	*AMERICAN JOURNAL OF CHINESE MEDICINE*	10	1.85%	4.833	Q1

The co-citation analysis from the Table 2 reveals a notable trend where journals with lower quartile rankings, such as Evidence-Based Complementary and Alternative Medicine [Q3, JIF (Journal Impact Factor) 2.650] and Acupuncture in Medicine (Q3, JIF 2.357), dominate the citation landscape with 63 and 21 citations, respectively. This indicates that while these journals are central to the field of EA for pain relief and emotional regulation, their lower impact factor may reflect a more limited influence outside the specialized area of complementary medicine. Conversely, higher-impact journals like International Journal of Molecular Sciences (Q1, JIF 4.860) and Brain Behavior and Immunity (Q1, JIF 8.814) are less frequently co-cited, with 23 and 14 citations respectively, suggesting that while they contribute significantly to the field, their influence is more selective.

**Table 2:** Top 10 co-cited journals.

Rank	Co-cited journal	Citations	JIF	Quartile in category (2024)
1	*EVIDENCE-BASED COMPLEMENTARY AND ALTERNATIVE MEDICINE*	63	2.650	Q3
2	*FRONTIERS IN NEUROSCIENCE*	30	3.202	Q2
3	*JOURNAL OF PAIN RESEARCH*	24	2.533	Q2
4	*INTERNATIONAL JOURNAL OF MOLECULAR SCIENCES*	23	4.860	Q1
5	*ACUPUNCTURE IN MEDICINE*	21	2.357	Q3
6	*AMERICAN JOURNAL OF CHINESE MEDICINE*	18	4.833	Q1
7	*NEURAL PLASTICITY*	17	3.106	Q3
8	*CHINESE JOURNAL OF INTEGRATIVE MEDICINE*	16	2.187	Q2
9	*BRAIN BEHAVIOR AND IMMUNITY*	14	8.814	Q1
10	*PLOS ONE*	13	2.928	Q2

### 3.6 Keywords

**Figure 5:**
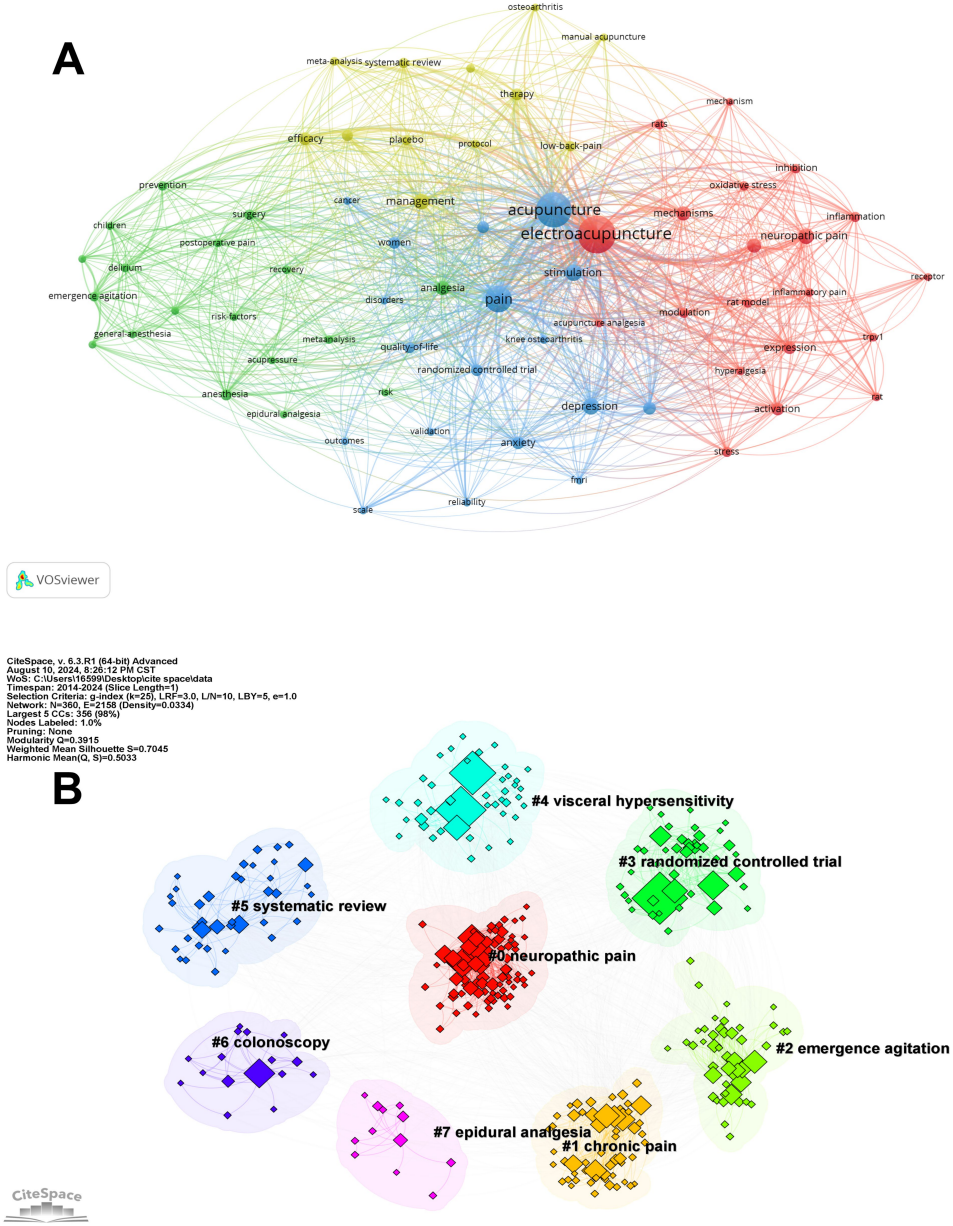
Keyword clusters. **(A)** The co-occurrence map of the keywords with high frequency. **(B)** Keyword clustering in EA’s analgesia and regulation on negative emotion.

Figure 5A offers a detailed visualization of keyword co-occurrence, with “acupuncture” and “EA” positioned as central nodes within a network created using VOSviewer. This network is divided into distinct clusters, each color-coded to represent different areas of research focus. For example, the red cluster predominantly explores the mechanisms through which acupuncture and EA influence pain, particularly in relation to neuropathic pain and inflammatory processes. The prominence of keywords like “oxidative stress” and “inflammation” within this cluster highlights a significant research interest in understanding the biochemical pathways involved in acupuncture’s analgesic effects. The blue cluster is centered on the broader application of acupuncture in pain management, including its impact on quality of life and its evaluation through randomized controlled trials. Meanwhile, the green cluster underscores the role of acupuncture in preventive care and perioperative settings, signifying its integration into comprehensive pain management strategies. The yellow cluster, on the other hand, reflects an emphasis on systematic reviews and meta-analyses, showcasing a growing trend toward consolidating and evaluating existing evidence to inform clinical practice.

Figure 5B, generated using CiteSpace, reveals several thematic clusters related to acupuncture’s application in pain relief and emotional regulation. Cluster #1, “chronic pain,” focuses on research exploring acupuncture’s role in managing persistent pain conditions. Cluster #2, “emergence agitation,” covers studies investigating acupuncture’s effects on reducing agitation and anxiety following anesthesia, linking closely to emotional regulation. Cluster #3, “randomized controlled trial,” highlights the use of rigorous clinical trials to establish the effectiveness of acupuncture in addressing both pain and emotional health. Cluster #4, “visceral hypersensitivity,” examines acupuncture’s potential in alleviating discomfort related to heightened sensitivity in internal organs, which can be associated with both physical pain and emotional distress. Cluster #5, “systematic review,” concentrates on compiling and analyzing existing research to provide an overview of acupuncture’s impact on pain management and emotional well-being. Cluster #6, “colonoscopy,” indicates a focus on reducing anxiety and discomfort through acupuncture during medical procedures. Finally, Cluster #7, “epidural analgesia,” looks at the combination of acupuncture with epidural analgesia, particularly during labor, to address both physical pain and the emotional aspects of childbirth.

## 4 Discussion

### 4.1 General information

According to results from the Web of Science database search, a total of 537 relevant articles attest to the burgeoning research fervor in this domain. These publications emanate from 485 authors representing 43 countries/regions, 20 institutions, and 353 journals. The analysis of annual publication trends shows a steady increase in research output, particularly after 2019. This suggests that EA has gained significant traction as a research area, likely due to its perceived effectiveness in treating these interconnected conditions. The surge in publications around 2019 aligns with a broader trend in alternative and complementary medicine, where increasing numbers of studies aim to provide scientific validation for traditional practices through rigorous methodologies.

Geographically, the research landscape is dominated by China, which accounts for a substantial portion of the total publications. This reflects China’s strong cultural and historical connection to acupuncture, coupled with significant governmental and institutional support for traditional Chinese medicine research. The Chinese government has actively promoted TCM as part of its healthcare strategy, providing funding and infrastructure for its integration with modern medical practices. Furthermore, China’s deep-rooted tradition in Chinese medicine and its ongoing emphasis on preserving and advancing these practices contribute to the country’s leadership in EA research. The contributions from other countries, including the United States, South Korea, and Taiwan, are also notable, although significantly smaller in comparison. This distribution indicates a global interest in EA, with China leading the charge, followed by contributions from countries with strong research traditions in complementary medicine.

Institutional analysis further highlights the dominance of Chinese institutions, with Zhejiang Chinese Medical University and Shanghai University of Traditional Chinese Medicine emerging as key players. These institutions have consistently produced a high volume of research, reflecting their deep commitment to advancing the study of EA. The collaborative networks revealed by the analysis indicate that while there is substantial cooperation within China, there are also meaningful collaborations with international institutions, albeit to a lesser extent. This suggests a growing, though still limited, global cooperation in advancing EA research. The prominence of Chinese institutions in both the volume and impact of research underscores China’s leadership in this field, while the involvement of institutions from the United States and South Korea signals a broadening interest and investment in EA from other parts of the world.

The analysis of authorship reveals a collaborative research community with over 485 contributors driving the field of EA for pain management and emotional regulation. Key figures include Fang Jianqiao, who has extensively studied the neurobiological mechanisms of EA. His collaborations with Du Junying, focusing on clinical applications of EA for chronic pain (21), and Shen Zui, exploring its psychological effects, highlight the multidisciplinary nature of the field (22). Emerging researchers like He Xiaofen and Shao Xiaomei are also making significant contributions, with He examining neurotransmitter regulation (23) and Shao investigating EA’s potential in managing anxiety and depression (24). The co-citation analysis underscores the influence of foundational researchers such as Han Jisheng, who pioneered the study of EA’s pain-relieving effects, and Zhang Ruixin, known for his work on EA’s impact on chronic pain through rat models. Together, these authors and their interconnected research efforts illustrate the dynamic and evolving landscape of EA studies.

When examining the journals in which the research about EA’s analgesia and regulation on negative emotion is published, the analysis shows a concentration of work in mid-impact journals, particularly those in the Q3 quartile. This suggests that while EA is gaining recognition, it has not yet fully penetrated the highest-impact journals in the broader medical and scientific communities. The predominance of specialized journals in complementary and alternative medicine within the most-cited sources reflects the niche, yet significant, influence of this research area. One potential reason for this is the highly specialized nature of EA research, which may not yet appeal to the broader, more general readership of high-impact journals. Many top-tier journals tend to focus on research with wide applicability across diverse medical fields, while EA research often addresses specific treatment methods or patient populations, limiting its fit with more generalist, high-impact journals. It also points to the challenges that remain in achieving wider acceptance and recognition of EA research within mainstream medical science, where higher-impact journals typically command more attention and credibility. To enhance the visibility of EA research in higher-impact journals, researchers must address methodological limitations, such as small sample sizes and the lack of large-scale randomized controlled trials (RCTs) and improve study quality through more rigorous experimental designs. Additionally, fostering interdisciplinary collaborations with fields like neurology and pharmacology can strengthen the scientific foundation of EA. Engaging more actively in clinical trials, publishing systematic reviews, and participating in guideline development can also help position EA as a credible, evidence-based therapy within conventional medical practice. While challenges remain, the steady presence of EA research in specialized journals lays a solid foundation for future growth and wider acceptance in high-impact publications.

### 4.2 Research hotspots and Frontiers

By conducting keyword co-occurrence analysis, we have identified primary hotspot areas in contemporary research about EA’s analgesia and regulation on negative emotion and proposed potential directions for future inquiry. A total of 360 keywords were incorporated into our statistical analysis, delineating several pivotal research themes. Integrating findings from two software programs, we have selected the most frequently encountered and research-significant keywords for comprehensive analysis.

#### 4.2.1 The role of the MAPK pathway

**Figure 6:**
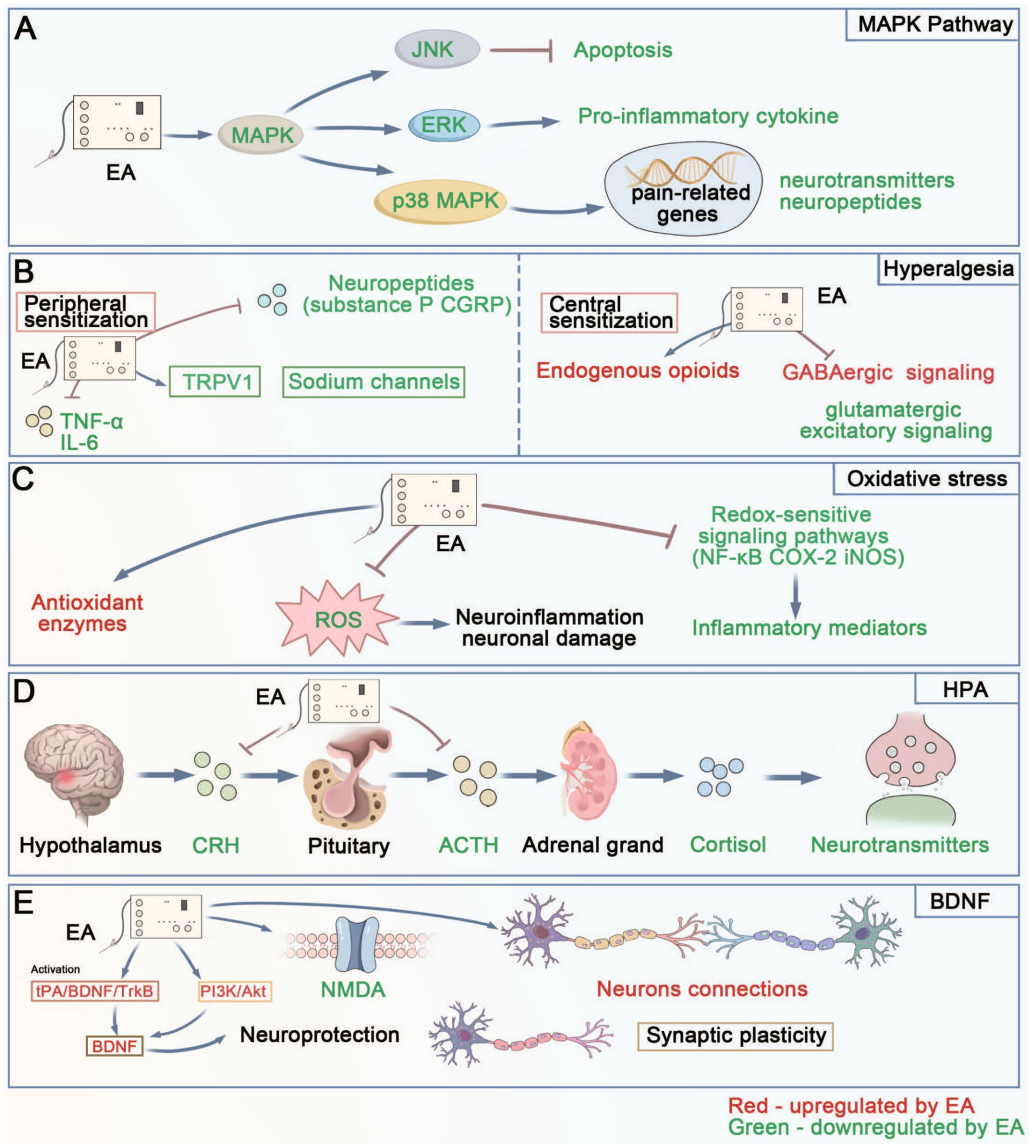
The diagram of the main mechanisms by which EA’s analgesia and regulation on negative emotion. **(A)** MAPK Pathway: Shows how EA regulates the MAPK pathway, where EA downregulates JNK (reducing apoptosis), ERK (decreasing pro-inflammatory cytokines), and p38 MAPK (affecting pain-related genes and neurotransmitter/neuropeptide expression). **(B)** Hyperalgesia: Illustrates EA’s effects on both peripheral and central sensitization. In peripheral sensitization, EA modulates TRPV1, sodium channels, and inflammatory factors (TNF-*α*, IL-6). In central sensitization, EA affects endogenous opioids, GABAergic signaling, and glutamatergic excitatory signaling. **(C)** Oxidative Stress: Depicts how EA regulates oxidative stress by enhancing antioxidant enzymes and suppressing ROS, which leads to reduced neuroinflammation and neuronal damage through modulation of redox-sensitive signaling pathways. **(D)** HPA Axis: Shows the sequential regulation of the HPA axis by EA, from CRH to ACTH to adrenal gland (cortisol), ultimately affecting neurotransmitter levels. **(E)** BDNF: Demonstrates EA’s effects on neuroplasticity through BDNF signaling, involving tPA/BDNF/TrkB and PI3K/Akt pathways, NMDA receptors, and neuronal connections, leading to neuroprotection and synaptic plasticity. MAPK, Mitogen-Activated Protein Kinase; JNK, c-Jun N-terminal Kinase; ERK, Extracellular Signal-Regulated Kinase; TRPV1, Transient Receptor Potential Vanilloid; TNF-α, Tumor Necrosis Factor-alpha; IL-6, Interleukin-6; CGRP, Calcitonin Gene-Related Peptide; ROS, Reactive Oxygen Species; NF-κB, Nuclear Factor kappa-B; COX-2, Cyclooxygenase-2; iNOS, inducible Nitric Oxide Synthase; HPA, Hypothalamic–Pituitary–Adrenal; CRH, Corticotropin-Releasing Hormone; ACTH, Adrenocorticotropic Hormone; BDNF, Brain-Derived Neurotrophic Factor; tPA, tissue Plasminogen Activator; TrkB, Tyrosine kinase receptor B; PI3K, Phosphatidylinositol 3-Kinase; Akt, Protein Kinase B; NMDA, N-Methyl-D-Aspartate.

The mitogen-activated protein kinase (MAPK) pathway plays a pivotal role in the mechanisms underlying EA-induced analgesia and emotional regulation (Figure 6A). The MAPK pathway, which includes subfamilies such as extracellular signal-regulated kinase (ERK), p38 MAPK, and c-Jun N-terminal kinase (JNK), is activated in response to EA, leading to the modulation of pain signaling pathways (25). Specifically, the activation of ERK and p38 MAPK influences the expression of pain-related genes and regulates the release of neurotransmitters and neuropeptides (26). This regulation is crucial for reducing inflammation and nociceptive sensitization, contributing to the analgesic effects of EA. EA inhibits ERK activation, thereby reducing pro-inflammatory cytokines, which helps alleviate pain and inflammation (27). Furthermore, the modulation of JNK activity by EA can influence the expression of genes associated with apoptosis and cell survival, providing neuroprotection and reducing neuropathic pain (28). In chronic pain conditions, persistent activation of MAPK pathways can lead to changes in neural plasticity, which may exacerbate pain perception. By modulating these pathways, EA helps restore normal neural function, thereby alleviating chronic pain (29). Additionally, the MAPK pathway’s involvement in cellular stress responses suggests that it plays a role in the adaptive response to pain, enhancing the body’s ability to cope with chronic pain conditions and promoting long-term analgesic effects (30).

In the context of emotional regulation, the MAPK pathway contributes significantly to the therapeutic effects of EA on mood disorders such as anxiety and depression, which frequently accompany chronic pain. EA-mediated suppression of MAPK pathway modulates neurotransmitter systems, including serotonin and norepinephrine, which are critical in mood regulation and neural plasticity (31). The enhancement of ERK signaling has been associated with increased neurogenesis and synaptic plasticity in brain regions implicated in mood regulation, such as the hippocampus. By promoting these neuroadaptive processes, EA can improve mood and cognitive function (32). The inhibition of stress-responsive kinases such as p38 MAPK, which are associated with anxiety and depression, supports EA’s role in emotional regulation (33).

#### 4.2.2 Hyperalgesia

Hyperalgesia, characterized by an increased sensitivity to pain, is a common phenomenon observed in various chronic pain conditions (34) (Figure 6B). EA has been found to effectively reduce hyperalgesia by modulating both peripheral and central mechanisms of pain processing (35). At the peripheral level, EA decreases the sensitivity of nociceptors—sensory neurons responsible for detecting harmful stimuli—through the regulation of ion channels such as TRPV1 and sodium channels (36). By modulating these ion channels, EA reduces the excitability of nociceptors, thereby decreasing pain perception (37). Additionally, EA can reduce the release of pro-inflammatory cytokines like TNF-*α*, IL-1β, and IL-6, which are known to enhance pain sensitivity by sensitizing nociceptors. This anti-inflammatory effect is crucial in chronic pain conditions, where ongoing inflammation contributes to sustained hyperalgesia (38). In chronic pain models, EA has demonstrated the ability to downregulate neuropeptides such as substance P and calcitonin gene-related peptide (CGRP), both of which are involved in the development and maintenance of hyperalgesia (39). The reduction in these pro-nociceptive substances not only diminishes pain transmission but also limits the inflammatory response, further alleviating pain.

At the central level, EA influences pain perception by modulating neurotransmitter systems in the spinal cord and brain, which are key sites for the integration and processing of pain signals. One of the primary mechanisms involves the increased release of endogenous opioids, such as endorphins, enkephalins, and dynorphins, during EA treatment. These opioids bind to and activate opioid receptors (e.g., mu, delta, and kappa receptors) in the central nervous system, inhibiting the transmission of pain signals and producing analgesic effects. The enhancement of the opioid system by EA not only provides immediate pain relief but also has long-term benefits by reducing central sensitization—a condition where the central nervous system exhibits heightened sensitivity to pain due to repeated or prolonged pain exposure (40). Furthermore, EA’s influence on neurotransmitter systems, including the enhancement of GABAergic (inhibitory) signaling and the reduction of glutamatergic (excitatory) signaling, further supports its ability to decrease central sensitization and hyperalgesia (41, 42). These combined actions not only alleviate pain but also address the emotional distress often associated with chronic pain conditions, such as anxiety and depression. By simultaneously targeting both the physical and psychological aspects of pain, EA provides a comprehensive therapeutic approach, demonstrating its dual benefits in managing chronic pain and improving overall quality of life for affected individuals.

#### 4.2.3 Oxidative stress

Oxidative stress is increasingly recognized as a significant factor in the therapeutic effects of EA for pain relief and emotional regulation (Figure 6C). This physiological condition, characterized by an imbalance between the production of reactive oxygen species (ROS) and the body’s antioxidant defenses, can lead to cellular damage, inflammation, and exacerbation of chronic pain (43). In chronic pain conditions, elevated ROS levels contribute to the sensitization of nociceptors and the amplification of pain signals (44). EA has been shown to help restore the oxidative balance by enhancing the activity of key antioxidant enzymes such as superoxide dismutase (SOD), catalase, and glutathione peroxidase (45). The reduction of ROS levels through EA not only protects cells from oxidative damage but also inhibits the activation of redox-sensitive signaling pathways, including the nuclear factor kappa-light-chain-enhancer of activated B cells (NF-κB) pathway. By mitigating the activation of NF-κB and related pathways, EA helps to reduce the expression of inflammatory mediators, thereby diminishing nociceptive signaling and alleviating pain. Additionally, EA’s modulation of oxidative stress is linked to its ability to suppress other pro-inflammatory pathways, such as those involving cyclooxygenase-2 (COX-2) and inducible nitric oxide synthase (iNOS), further contributing to its analgesic effects (46, 47).

Beyond pain relief, the impact of oxidative stress on mental health highlights its role in EA’s benefits for emotional well-being. Elevated ROS levels are associated with neuroinflammation, disrupted neurotransmitter signaling, and neuronal damage, all of which are implicated in the pathophysiology of mood disorders such as anxiety and depression (48). Chronic oxidative stress can impair the function of neurotransmitters like serotonin, dopamine, and norepinephrine, which are crucial for maintaining mood stability and cognitive function (49). EA’s ability to reduce oxidative stress helps to protect neuronal integrity by enhancing mitochondrial function, reducing neuroinflammation, and preserving the structure of synapses, which are essential for effective neurotransmission. The reduction in oxidative stress achieved through EA not only alleviates the biochemical disruptions associated with mood disorders but also helps to restore normal brain function, thereby offering a holistic approach to managing the emotional and psychological aspects of chronic pain.

#### 4.2.4 The hypothalamic–pituitary–adrenal axis

The hypothalamic–pituitary–adrenal (HPA) axis is a central stress response system that plays a significant role in both analgesia and emotional regulation (50), making it a critical area of focus in the study of EA effects (Figure 6D). The HPA axis is activated in response to stress, initiating a cascade that begins with the hypothalamus releasing CRH (Corticotropin releasing hormone). CRH then stimulates the anterior pituitary to secrete ACTH (adrenocorticotropic hormone), which subsequently triggers the adrenal glands to release cortisol, a key stress hormone (51). Elevated cortisol levels are associated with numerous adverse effects, including increased inflammation, immune suppression, and heightened pain sensitivity (52). EA modulates the HPA axis by regulating the release of CRH and ACTH, leading to a decrease in cortisol production (53). This reduction in cortisol helps to attenuate the body’s stress response, thereby alleviating symptoms of anxiety and depression, which are prevalent in chronic pain patients. The downregulation of CRH and ACTH through EA has also been linked to decreased activation of the sympathetic nervous system, resulting in lower levels of norepinephrine and reduced sympathetic tone (54). This modulation helps improve pain management by reducing hyperarousal and the heightened pain sensitivity that often accompanies chronic stress.

Moreover, the interaction between the HPA axis and other neurotransmitter systems highlights EA’s complex regulatory role in stress and pain management. Chronic stress can disrupt the balance of neurotransmitters, leading to alterations in mood, behavior, and pain perception. EA’s effects on the HPA axis not only reduces cortisol levels but also modulates the release of key neurotransmitters, such as serotonin, dopamine, and norepinephrine, which are critical for emotional stability and pain perception (54). Additionally, the modulation of neurotransmitter systems by EA can positively influence motivation and reward processing, which are often impaired in individuals suffering from chronic pain and depression (55). EA’s normalization of HPA axis activity also helps to maintain the balance between excitatory and inhibitory neurotransmitters, restoring the homeostatic balance that is often disrupted by chronic stress. This balanced state supports the brain’s natural ability to cope with stress and reduces the risk of developing stress-related disorders.

#### 4.2.5 Brain-derived neurotrophic factor

Brain-derived neurotrophic factor (BDNF) is a key neurotrophin involved in the survival, growth, and maintenance of neurons, as well as in synaptic plasticity—the ability of synapses to strengthen or weaken over time, which is essential for learning and memory (56) (Figure 6E). In the context of pain modulation, BDNF plays a vital role in both the peripheral and central nervous systems. Research indicates that EA can significantly increase BDNF levels in critical pain-related regions of the brain, such as the spinal cord, periaqueductal gray matter, and even the hippocampus (57–59). This increase in BDNF is associated with the activation of intracellular signaling pathways like tPA (tissue plasminogen activator)/BDNF/TrkB (tyrosine kinase receptor B) and PI3K/Akt (phosphoinositide 3-kinase/Akt), which are involved in cellular survival, neuroprotection, and synaptic plasticity (60, 61). By activating these pathways, EA enhances the expression of genes that promote neuronal survival and inhibit apoptosis, thereby reducing pain sensitivity and improving the body’s natural pain control mechanisms. Furthermore, BDNF can modulate the expression of ion channels and neurotransmitter receptors, such as the N-methyl-D-aspartate (NMDA) receptor, which are critical in the transmission of pain signals (62). Through these mechanisms, EA-induced upregulation of BDNF contributes to the reduction of hyperalgesia (increased sensitivity to pain) and allodynia (pain from normally non-painful stimuli), common features of chronic pain conditions.

In addition to its role in pain relief, BDNF is also deeply involved in regulating emotional responses, making it highly relevant for addressing the psychological aspects of chronic pain. Chronic pain is often accompanied by mood disorders such as anxiety and depression, conditions in which BDNF levels are typically found to be reduced (63). By enhancing BDNF expression, EA not only alleviates pain but also exerts antidepressant-like effects. Increased BDNF levels have been shown to promote neurogenesis, particularly in the hippocampus, a brain region implicated in mood regulation and cognitive function. This neurogenic effect is crucial for combating the neurodegenerative aspects of depression and anxiety (64). Additionally, BDNF enhances synaptic plasticity, which strengthens the connections between neurons, thereby improving the brain’s capacity to adapt to stress and maintain emotional stability (65). Through these interactions, EA-induced BDNF expression not only helps improve mood but also supports overall emotional resilience.

## 5 Limitations

This bibliometric analysis has some limitations. Firstly, it primarily relies on the Web of Science database, which may not cover all relevant studies, especially those published in non-English journals or regional databases. Secondly, the study focuses on publications from 2014 to 2024, potentially missing out on earlier influential research. Thirdly, citation biases, such as self-citations, could affect the results. Future research should address these issues to provide a more comprehensive understanding. Lastly, this analysis emphasizes quantitative data over qualitative insights, which limits the understanding of the methodological rigor and clinical relevance of the included studies.

## 6 Conclusion

The bibliometric analysis of EA for analgesia and emotional regulation from 2014 to 2024 reveals a significant rise in research activity, especially since 2019, reflecting a growing global interest in its therapeutic potential. This increase aligns with the broader trend of validating traditional therapies through scientific research, marking EA’s integration into modern medical practice. China, with its strong cultural and historical connection to acupuncture and substantial institutional support for traditional Chinese medicine, leads the field, though significant contributions from the United States and South Korea indicate expanding international interest. Key research themes include neurotransmitter modulation, endogenous opioid release, and EA’s application in managing chronic pain and mood disorders, showcasing a multidisciplinary approach combining neurobiology, psychology, and traditional medicine. Despite its prominence, EA research is predominantly published in mid-impact journals and faces challenges in achieving broader acceptance in higher-impact medical journals. To enhance the field’s scientific standing, future research should focus on elucidating the specific mechanisms of EA’s therapeutic effects, exploring its efficacy in diverse clinical settings, and fostering international collaboration. These efforts will help establish EA as a valuable approach for managing complex physical and emotional conditions.

## Data Availability

The original contributions presented in the study are included in the article, further inquiries can be directed to the corresponding author.

## References

[1] Zhang R Lao L Ren K Berman BM. Mechanisms of acupuncture-electroacupuncture on persistent pain. Anesthesiology. (2014) 120:482–503. doi: 10.1097/ALN.0000000000000101 24322588 PMC3947586

[2] Amorim D Amado J Brito I Fiuza SM Amorim N Costeira C . Acupuncture and electroacupuncture for anxiety disorders: a systematic review of the clinical research. Complement Ther Clin Pract. (2018) 31:31–7. doi: 10.1016/j.ctcp.2018.01.008 29705474

[3] Zhou Z Xu G Huang L Tian H Huang F Liu Y . Effectiveness and safety of electroacupuncture for depression: a systematic review and meta-analysis. Evid Based Complement Altern Med. (2022) 2022:1–15. doi: 10.1155/2022/4414113 PMC9410808 36034955

[4] Woolf CJ. What is this thing called pain? J Clin Invest. (2010) 120:3742–4. doi: 10.1172/JCI45178 21041955 PMC2965006

[5] Michaelides A Zis P. Depression, anxiety and acute pain: links and management challenges. Postgrad Med. (2019) 131:438–44. doi: 10.1080/00325481.2019.1663705 31482756

[6] Kaptchuk TJ. Acupuncture: theory, efficacy, and practice. Ann Intern Med. (2002) 136:374–83. doi: 10.7326/0003-4819-136-5-200203050-00010 11874310

[7] Zhu W Jia Q Ferreira AC Jiang H Zhang J Li B . Acupuncture for ischemic stroke: where are we now? Acupunct Herb Med. (2024) 4:36. doi: 10.1097/HM9.0000000000000094

[8] Guo Z Wei N Ye R Sun T Qiu S Shao X . Map activation of various brain regions using different frequencies of electroacupuncture ST36, utilizing the FosCreER strategy. Acupunct Herb Med. (2024) 4:386–98. doi: 10.1097/HM9.0000000000000106

[9] Shi JT Cao WY Zhang XN Wan HY Su YS Qu ZY . Local analgesia of electroacupuncture is mediated by the recruitment of neutrophils and released β-endorphins. Pain. (2023) 164:1965–75. doi: 10.1097/j.pain.0000000000002892 37027145 PMC10436362

[10] Li R Sun J Luo K Luo N Sun R Gao F . Electroacupuncture and carbamazepine for patients with trigeminal neuralgia: a randomized, controlled, 2 × 2 factorial trial. J Neurol. (2024) 271:5122–36. doi: 10.1007/s00415-024-12433-x 38816482 PMC11319385

[11] Wu SY Lin CH Chang NJ Hu WL Hung YC Tsao Y . Combined effect of laser acupuncture and electroacupuncture in knee osteoarthritis patients: a protocol for a randomized controlled trial. Medicine (Baltimore). (2020) 99:e19541. doi: 10.1097/MD.0000000000019541 32195960 PMC7220484

[12] Hsiao IH Lin YW. Electroacupuncture reduces fibromyalgia pain by attenuating the HMGB1, S100B, and TRPV1 Signalling pathways in the mouse brain. Evid Based Complement Altern Med. (2022) 2022:2242074. doi: 10.1155/2022/2242074 PMC8941543 35341159

[13] Ahn J-H Song M-Y Park H-J. Discovering influential Core-keywords, researcher networks and research trends of Acupuncture on depression using bibliometric analysis. J Acupunct Meridian Stud. (2022) 15:227–37. doi: 10.51507/j.jams.2022.15.4.227 36521771

[14] Li T Yan QL Huang W. Research trends on acupuncture for neuropathic pain: a bibliometric analysis from 1979 to 2023. Medicine. (2024) 103:e37962. doi: 10.1097/MD.0000000000037962 38701301 PMC11062671

[15] Li ZQ Wang XF Feng C Fei YT Liu JP. Global trends of acupuncture clinical research on analgesia from 2010 to 2023: a bibliometric and visualization analysis. Front Neurol. (2024) 15:1368988. doi: 10.3389/fneur.2024.1368988 38665996 PMC11043534

[16] Liu D Chen B Li T Zheng LJ Li JL Du WY . Research hotspots and trends on Acupuncture for neuropathic pain: a bibliometric analysis from 2002 to 2021. J Pain Res. (2022) 15:3381–97. doi: 10.2147/JPR.S383291 36317163 PMC9617558

[17] Yang P Wang T He YJ Su SY. Research trends of Acupuncture therapy for chronic pain-related depression or anxiety from 2003 to 2023: a bibliometric analysis. J Pain Res. (2023) 16:4301–15. doi: 10.2147/JPR.S436434 38116394 PMC10729835

[18] Ulett GA Han S Han JS. Electroacupuncture: mechanisms and clinical application. Biol Psychiatry. (1998) 44:129–38. doi: 10.1016/S0006-3223(97)00394-6 9646895

[19] Zhang RX Li A Liu B Wang L Xin J Ren K . Electroacupuncture attenuates bone-cancer-induced hyperalgesia and inhibits spinal preprodynorphin expression in a rat model. Eur J Pain. (2008) 12:870–8. doi: 10.1016/j.ejpain.2007.12.006 18221900 PMC3107701

[20] Vickers AJ Cronin AM Maschino AC Lewith G MacPherson H Foster NE . Acupuncture for chronic pain: individual patient data meta-analysis. Arch Intern Med. (2012) 172:1444–53. doi: 10.1001/archinternmed.2012.3654 22965186 PMC3658605

[21] Wu M Chen Y Shen Z Zhu Y Xiao S Zhu X . Electroacupuncture alleviates anxiety-like behaviors induced by chronic neuropathic pain via regulating different dopamine receptors of the basolateral amygdala. Mol Neurobiol. (2022) 59:5299–311. doi: 10.1007/s12035-022-02911-6 35696012 PMC9395447

[22] Wu Z Shen Z Xu Y Chen S Xiao S Ye J . A neural circuit associated with anxiety-like behaviors induced by chronic inflammatory pain and the anxiolytic effects of electroacupuncture. CNS Neurosci Ther. (2024) 30:e14520. doi: 10.1111/cns.14520 38018559 PMC11017463

[23] Li X Zhu Y Sun H Shen Z Sun J Xiao S . Electroacupuncture inhibits pain memory and related anxiety-like behaviors by blockading the GABAB receptor function in the midcingulate cortex. Mol Neurobiol. (2023) 60:6613–26. doi: 10.1007/s12035-023-03467-9 37468738 PMC10533721

[24] Li Y Liu X Fu Q Fan W Shao X Fang J . Electroacupuncture ameliorates depression-like behaviors comorbid to chronic neuropathic pain via Tet1-mediated restoration of adult neurogenesis. Stem Cells Dayt Ohio. (2023) 41:384–99. doi: 10.1093/stmcls/sxad007 36648299

[25] Fang M Lan Y Li M Li C Xu B Ma Y . Electroacupuncture targeting the immune system to alleviate sepsis. Acupunct Herb Med. (2024) 4:56–67. doi: 10.1097/HM9.0000000000000092

[26] Zhen W Zhen H Wang Y Chen L Niu X Zhang B . Mechanism of ERK/CREB pathway in pain and analgesia. Front Mol Neurosci. (2023) 16:1156674. doi: 10.3389/fnmol.2023.1156674 37008781 PMC10060514

[27] Li SS Tu WZ Zhong YB Jiang X Jiang SH. Involvement of central neuroglia cells in analgesic effect of electroacupuncture therapy for neuropathic pain. Zhen Ci Yan Jiu. (2016) 41:369–72. 29071937

[28] Chen T Zhang WW Chu YX Wang YQ. Acupuncture for pain management: molecular mechanisms of action. Am J Chin Med. (2020) 48:793–811. doi: 10.1142/S0192415X20500408 32420752

[29] Zhao ZQ. Neural mechanism underlying acupuncture analgesia. Prog Neurobiol. (2008) 85:355–75. doi: 10.1016/j.pneurobio.2008.05.004 18582529

[30] Hotamisligil GS Davis RJ. Cell signaling and stress responses. Cold Spring Harb Perspect Biol. (2016) 8:a006072. doi: 10.1101/cshperspect.a006072 27698029 PMC5046695

[31] Wang JQ Mao L. The ERK pathway: molecular mechanisms and treatment of depression. Mol Neurobiol. (2019) 56:6197–205. doi: 10.1007/s12035-019-1524-3 30737641 PMC6684449

[32] Marsden WN. Synaptic plasticity in depression: molecular, cellular and functional correlates. Prog Neuro-Psychopharmacol Biol Psychiatry. (2013) 43:168–84. doi: 10.1016/j.pnpbp.2012.12.012 23268191

[33] Li X Teng T Yan W Fan L Liu X Clarke G . AKT and MAPK signaling pathways in hippocampus reveals the pathogenesis of depression in four stress-induced models. Transl Psychiatry. (2023) 13:200. doi: 10.1038/s41398-023-02486-3 37308476 PMC10261007

[34] Stoicea N Russell D Weidner G Durda M Joseph NC Yu J . Opioid-induced hyperalgesia in chronic pain patients and the mitigating effects of gabapentin. Front Pharmacol. (2015) 6:104. doi: 10.3389/fphar.2015.00104 26074817 PMC4444749

[35] Ma X Chen W Fu Y Li H Liu C. Acupuncture for neuropathic pain: focusing on the sympathetic nerve system. Acupunct Herb Med. (2023) 3:139. doi: 10.1097/HM9.0000000000000069

[36] Guo Y Li Y Xu T Zhu MX Xu Z Dou B . An inspiration to the studies on mechanisms of acupuncture and moxibustion action derived from 2021 Nobel prize in physiology or medicine. Acupunct Herb Med. (2022) 2:1–8. doi: 10.1097/HM9.0000000000000023

[37] Liu Y Du J Fang J Xiang X Xu Y Wang S . Electroacupuncture inhibits the interaction between peripheral TRPV1 and P2X3 in rats with different pathological pain. Physiol Res. (2021) 70:635–47. doi: 10.33549/physiolres.934649, PMID: 34062076 PMC8820540

[38] Qu SY Wang HZ Hu QQ Ma YQ Kang YR Ma LQ . Electroacupuncture may alleviate diabetic neuropathic pain by inhibiting the microglia P2X4R and neuroinflammation. Purinergic Signal. (2023). doi: 10.1007/s11302-023-09972-9 PMC12454746 37870716

[39] Zhang RY Zhu BF Wang LK Song Y Zhao JG Guo Y . Electroacupuncture alleviates inflammatory pain via adenosine suppression and its mediated substance P expression. Arq Neuropsiquiatr. (2020) 78:617–23. doi: 10.1590/0004-282x20200078 33146290

[40] Lin JG Chen WL. Acupuncture analgesia: a review of its mechanisms of actions. Am J Chin Med. (2008) 36:635–45. doi: 10.1142/S0192415X08006107 18711761

[41] Sun J Zhang C Wang Y Xiao S Sun H Bian Z . Electroacupuncture alleviates hyperalgesia and anxiety-like behaviors in pain memory model rats through activation of GABAergic neurons and GABA receptor in the rostral anterior cingulate cortex. Mol Neurobiol. (2024) 61:6613–27. doi: 10.1007/s12035-024-03986-z 38329679 PMC11338974

[42] Wang JY Zhang JL Chen SP Gao YH Zhang JL Chen Y . Electroacupuncture relieves hyperalgesia by regulating neuronal-glial interaction and glutamate transporters of spinal dorsal horns in rats with acute incisional neck pain. Front Neurosci. (2022) 16:885107. doi: 10.3389/fnins.2022.885107 36389227 PMC9643735

[43] Sies H. Oxidative stress: a concept in redox biology and medicine. Redox Biol. (2015) 4:180–3. doi: 10.1016/j.redox.2015.01.002 25588755 PMC4309861

[44] Assavarittirong C Samborski W Grygiel-Górniak B. Oxidative stress in fibromyalgia: from pathology to treatment. Oxidative Med Cell Longev. (2022) 2022:1–11. doi: 10.1155/2022/1582432 PMC9556195 36246401

[45] Zhang RY Zhu BF Zhao JG Zhao L Wang LK. Electroacupuncture stimulation alleviates inflammatory pain in male rats by suppressing oxidative stress. Physiol Res. (2023) 72:657–67. doi: 10.33549/physiolres.934965 38015764 PMC10751055

[46] Wang HF Chen L Xie Y Wang XF Yang K Ning Y . Electroacupuncture facilitates M2 macrophage polarization and its potential role in the regulation of inflammatory response. Biomed Pharmacother Biomedecine Pharmacother. (2021) 140:111655. doi: 10.1016/j.biopha.2021.111655 34029955

[47] Lee JH Jang KJ Lee YT Choi YH Choi BT. Electroacupuncture inhibits inflammatory edema and hyperalgesia through regulation of cyclooxygenase synthesis in both peripheral and central nociceptive sites. Am J Chin Med. (2006) 34:981–8. doi: 10.1142/S0192415X06004454 17163587

[48] Kalinichenko LS Kornhuber J Müller CP. Individual differences in inflammatory and oxidative mechanisms of stress-related mood disorders. Front Neuroendocrinol. (2019) 55:100783. doi: 10.1016/j.yfrne.2019.100783 31415777

[49] Belleau EL Treadway MT Pizzagalli DA. The impact of stress and major depressive disorder on hippocampal and medial prefrontal cortex morphology. Biol Psychiatry. (2019) 85:443–53. doi: 10.1016/j.biopsych.2018.09.031 30470559 PMC6380948

[50] Liu J Dong S Liu S. Aberrant parasympathetic responses in acupuncture therapy for restoring immune homeostasis. Acupunct Herb Med. (2023) 3:69–75. doi: 10.1097/HM9.0000000000000060

[51] Karaca Z Grossman A Kelestimur F. Investigation of the Hypothalamo-pituitary-adrenal (HPA) axis: a contemporary synthesis. Rev Endocr Metab Disord. (2021) 22:179–204. doi: 10.1007/s11154-020-09611-3 33770352

[52] Knezevic E Nenic K Milanovic V Knezevic NN. The role of cortisol in chronic stress, neurodegenerative diseases, and psychological disorders. Cells. (2023) 12:2726. doi: 10.3390/cells12232726 38067154 PMC10706127

[53] Zheng JY Zhu J Wang Y Tian ZZ. Effects of acupuncture on hypothalamic-pituitary-adrenal axis: current status and future perspectives. J Integr Med. (2024) 22:445–58. doi: 10.1016/j.joim.2024.06.004 38955651

[54] Han X Gao Y Yin X Zhang Z Lao L Chen Q . The mechanism of electroacupuncture for depression on basic research: a systematic review. Chin Med. (2021) 16:10. doi: 10.1186/s13020-020-00421-y 33436036 PMC7805231

[55] Yang M Baser RE Khanin R Autuori I Li QS Panageas KS . COMT Val158Met affects the analgesic response to Acupuncture among Cancer survivors with chronic pain. J Pain. (2023) 24:1721–30. doi: 10.1016/j.jpain.2023.05.005 37187218 PMC11321469

[56] Colucci-D'Amato L Speranza L Volpicelli F. Neurotrophic factor BDNF, physiological functions and therapeutic potential in depression, neurodegeneration and brain Cancer. Int J Mol Sci. (2020) 21:7777. doi: 10.3390/ijms21207777 33096634 PMC7589016

[57] Shin KM Ko IG Kim SE Jin JJ Hwang L Kim SH . Low-frequency electroacupncture improves locomotor function after sciatic crushed nerve injury in rats. J Exerc Rehabil. (2018) 14:927–33. doi: 10.12965/jer.1836594.297 30656150 PMC6323326

[58] Zhang BL Guo XL. Electroacupuncture promotes nerve regeneration and functional recovery in rats with spinal cord contusion through the coordinate interaction of CD4 and BDNF. Ibrain. (2022) 8:285–301. doi: 10.1002/ibra.12055 37786738 PMC10529162

[59] Pei W Meng F Deng Q Zhang B Gu Y Jiao B . Electroacupuncture promotes the survival and synaptic plasticity of hippocampal neurons and improvement of sleep deprivation-induced spatial memory impairment. CNS Neurosci Ther. (2021) 27:1472–82. doi: 10.1111/cns.13722 34623740 PMC8611786

[60] Dong H Qin YQ Sun YC Yao HJ Cheng XK Yu Y . Electroacupuncture ameliorates depressive-like behaviors in poststroke rats via activating the tPA/BDNF/TrkB pathway. Neuropsychiatr Dis Treat. (2021) 17:1057–67. doi: 10.2147/NDT.S298540 33880028 PMC8053498

[61] Chen A Lin Z Lan L Xie G Huang J Lin J . Electroacupuncture at the Quchi and Zusanli acupoints exerts neuroprotective role in cerebral ischemia-reperfusion injured rats via activation of the PI3K/Akt pathway. Int J Mol Med. (2012) 30:791–6. doi: 10.3892/ijmm.2012.1074, PMID: 22842715

[62] Ren K Dubner R. Pain facilitation and activity-dependent plasticity in pain modulatory circuitry: role of BDNF-TrkB signaling and NMDA receptors. Mol Neurobiol. (2007) 35:224–35. doi: 10.1007/s12035-007-0028-8 17917111

[63] Kraus C Kadriu B Lanzenberger R Zarate CA Kasper S. Prognosis and improved outcomes in major depression: a review. Transl Psychiatry. (2019) 9:127. doi: 10.1038/s41398-019-0460-3 30944309 PMC6447556

[64] Camuso S La Rosa P Fiorenza MT Canterini S. Pleiotropic effects of BDNF on the cerebellum and hippocampus: implications for neurodevelopmental disorders. Neurobiol Dis. (2022) 163:105606. doi: 10.1016/j.nbd.2021.105606 34974125

[65] Leal G Comprido D Duarte CB. BDNF-induced local protein synthesis and synaptic plasticity. Neuropharmacology. (2014) 76:639–56. doi: 10.1016/j.neuropharm.2013.04.005 23602987

